# Metabolomic Analysis, Combined with Enzymatic and Transcriptome Assays, to Reveal the Browning Resistance Mechanism of Fresh-Cut Eggplant

**DOI:** 10.3390/foods11081174

**Published:** 2022-04-18

**Authors:** Xiaohui Liu, Kai Xiao, Aidong Zhang, Weimin Zhu, Hui Zhang, Feng Tan, Qianru Huang, Xuexia Wu, Dingshi Zha

**Affiliations:** 1 Shanghai Key Laboratory of Protected Horticultural Technology, Horticultural Research Institute, Shanghai Academy of Agricultural Sciences, Shanghai 201403, China; liuxiaohui927@163.com (X.L.); 15562791500@163.com (K.X.); family20082008@sina.com (A.Z.); wmzhu69@126.com (W.Z.); zhanghui@saas.sh.cn (H.Z.); tfliujianfeng@gmail.com (F.T.); huangqianru1003@163.com (Q.H.); dingshizha@aliyun.com (D.Z.); 2 College of Food Science, Shanghai Ocean University, Shanghai 201306, China

**Keywords:** *Solanum melongena*, browning, transcriptome, metabolome, membrane lipid metabolism

## Abstract

Browning has been the primary limitation in eggplant processing. This study investigates the molecular mechanism underlying fresh-cut eggplant fruit browning by observing the physicochemical characteristics of browning-resistant (‘F’) and browning-sensitive (‘36′) eggplant cultivars. Browning-related enzyme activity and gene expression (PPO, LOX, and PLD) were significantly higher in the ‘36′ eggplant, thereby enhancing the degree of browning, compared to the ‘F’ eggplant. The MDA content and O_2_^−^ production rate progressively increased as browning increased, while the antioxidant capacity of the fruit decreased. The cutting injury significantly activated the expression of PAL, thereby inducing the accumulation of phenolic acids, while the PPO gene was significantly upregulated, which activated the activity of polyphenol oxidase. Our results showed that the oxidation of chlorogenic acids to chlorogenic quinones resulted in the occurrence of browning, which suggests chlorogenic acid as the main browning substrate in fresh-cut eggplant.

## 1. Introduction

Eggplant (*Solanum melongena* L.) is an important affordable vegetable worldwide, with a large cultivation area, rich nutritional value, and steady annual supply [1]. Eggplants often suffer serious mechanical damage during processing, resulting in fruit browning [2]. The browning of fruits and vegetables not only affects their sensory, nutritional, and edible qualities, but also reduces their economic value, hinders their distribution and consumption, and shortens their shelf life [3]. Enzymatic browning is thought to be the primary cause of fruit and vegetable browning during postharvest storage and processing, and it has long been the focus of postharvest research [4].

During enzymatic browning, phenolic substrates in fruit and vegetables are catalyzed by polyphenol oxidase (PPO) to form quinones, while the polymerized quinones rapidly become dark-colored pigments [5]. Tissue browning in harvested horticultural products has been linked to structural and functional damage to cellular membranes, according to previous research [6]. The cell membrane is an important structure of cells, which provides a stable internal environment for cellular activities and enables nutrient exchange and cell recognition [7]. Under normal physiological conditions, enzymes and substrates are regionally distributed in cells through a series of membrane systems. However, under external stress, such as abiotic stress, abnormal physiological changes occur, thereby breaking the localized distribution and leading to a direct contact between PPO and substrates, followed by enzymatic browning [8]. Thus, in essence, fruit and vegetable browning is the disintegration of the cell membrane structure and disruption of the normal distribution of enzymes and substrates, resulting in the enzymatic oxidation of phenol substances.

Reactive oxygen species (ROS) metabolism is necessary for enzymatic browning. The production and clearance of ROS is in dynamic equilibrium; however, if this balance is disrupted, ROS accumulate, and a large number of free radicals initiate membrane lipid peroxidation, destroying the membrane structure and stimulating the catabolism of membrane lipids [9]. Phospholipids are the main structural components of the cell membrane and play an important role in maintaining cell structure, protein function, and signal transduction [7]. Unsaturated fatty acids (USFAs) maintain the unsaturated and fluid nature of cell membranes, which is necessary for plants to survive under various stresses [10]. A previous study found that the browning of ‘Nanguo’ pears was linked to cellular membrane system damage, such as phospholipid degradation and USFA peroxidation [11]. Phospholipase D (PLD), lipoxygenase (LOX), and lipase are involved in the oxidation of membrane lipids, according to another study [12]. An intermittent warming treatment reduced the enzyme activity and gene expression of PLD and LOX, which are involved in glycerophospholipid metabolism, thereby alleviating the browning of the ‘Nanguo’ pear peel [12].

Meanwhile, in fresh eggplant, browning is also attributed to the effect on both phenolic content and PPO-specific activity, whereas total phenolic content played a prominent part in the browning of stored fruits [2]. Previous findings have suggested that increasing the content of phenolic acids, especially chlorogenic acid, in eggplant flesh increases the sensitivity of eggplant flesh to browning [13]. However, In the studied genetic lines, a significant correlation has been reported between chlorogenic acid and total phenols, but no correlation between flesh browning and high chlorogenic acid content or increased PPO activity. The authors hypothesize that the difficult and complicated mechanism of flesh browning in eggplant genetic lines is mediated by a number of distinct factors [14]. In this study, the phenotypes and key enzyme activities of fresh-cut eggplant fruits were analyzed. Transcriptomic and metabolomic analyses were used to determine the changes in the membrane phospholipid and unsaturated fatty acid metabolite content and gene expression related to membrane lipid metabolism. This study investigated the mechanism underlying browning in fresh-cut eggplant fruit from the standpoint of membrane lipid metabolism. Our results provide insights into a foundation for further research into the processes that regulate fruit browning in fresh-cut fruit and vegetables.

## 2. Materials and Methods

### 2.1. Plant Material and Treatments

The eggplant cultivars ‘36′ (browning-sensitive) and ‘F’ (browning-resistant) were collected from a breeding line developed in our laboratory at the Shanghai Academy of Agricultural Sciences’ Shanghai Key Laboratory of Protected Horticultural Technology in Shanghai, China. For the experiment, mature commercial eggplant fruits with the same growth trend and uniform size and with no diseases, insect pests, or obvious mechanical damage were selected. Using a stainless-steel knife, the fruit samples were carefully peeled. Based on the time since cutting, three treatments groups for ‘36′ and ‘F’ were established: control, fresh-cut for 0 min (36 CK0 and F CK0, respectively); fresh-cut for 2 min (36 2 min and F 2 min, respectively); fresh-cut for 15 min (36 15 min and F 15 min, respectively). The samples were frozen and stored at −80 °C until RNA extraction. For the analysis, six independent replicates were used.

### 2.2. Browning Degree

Browning Degree (BD) was determined according to the procedure of Kan et al. [15] with some modifications. Briefly, a 1.0 g eggplant sample was homogenized in 10 mL precooled distilled water and centrifuged (4 °C, 12,000 rpm, 10 min). The supernatant was collected, and the absorption value at 410 nm was recorded using distilled water as the control. BD was expressed as 10 × A410 nm.

### 2.3. Metabolite Identification and Statistical Analysis

The sample extracts were analyzed via a LC-MS/MS system (ACQUITY UPLC I-Class system (Waters Corporation, Milford, MA, USA) coupled with VION IMS QTOF Mass spectrometer (Waters Corporation, Milford, MA, USA). Progenesis QI v2.3 was used for baseline filtering, peak identification, integral, retention time, alignment, calibration peak, and normalization (Nonlinear Dynamics, Newcastle, UK). The Human Metabolome Database (HMDB), Lipidmaps (V2.3), and METLIN Database were used to identify compounds based on reliable mass number secondary fragments and isotope distribution. Unsupervised principal component analysis (PCA) was used in multivariate statistical analysis to determine the overall distribution of each sample and the stability of the entire analysis process, followed by supervised partial least squares analysis (PLS-DA) and orthogonal partial least squares analysis (OPLS-DA) to determine the difference in metabolites between groups. The variable influence of projection (VIP) > 1 for the first principal component in the OPLS-DA and *p*-value < 0.05 were used to screen differential metabolites. The KEGG database was used to conduct a metabolic pathway enrichment analysis of different metabolites in different samples.

### 2.4. Integrated Analysis of the Metabolome and Transcriptome

Combined with the published transcriptome data of fresh-cut eggplant [16] (SRA: PRJNA679701 (http://www.ncbi.nlm.nih.gov/sra, accessed on 20 November 2020)), the EXCEL program was used to calculate the Pearson correlation coefficients according to the fold changes of each DEG and DEM. Correlations corresponding to a coefficient with R^2^ > 0.8 were selected, and the co-expression pathway of the transcriptome and metabolome was constructed.

### 2.5. Membrane Lipid Peroxidation (MDA, H_2_O_2_, and O_2_^−^)

Malondialdehyde, H_2_O_2_, and O_2_^−^ production rates were determined using an appropriate assay kit according to the manufacturer’s instructions (Suzhou Grace Biotechnology Co., Ltd., Suzhou, China). Briefly, fresh eggplant samples (0.2 g) were homogenized in an extraction liquid (1:5, *v*/*v*). The difference of absorbance between 532 and 600 nm was used to calculate the content of MDA. The content of H_2_O_2_ determined via absorbance measurements at 415 nm. The readings at 530 nm were also recorded for the O_2_^−^ production rate and were expressed as nanomolar per gram per minute (nmol g^−1^ min^−1^).

### 2.6. Activities of PPO, LOX, PLD, PAL, CAT, SOD, POD, and APX

The activities of PPO, LOX, PLD, PAL, CAT, SOD, POD, and APX were determined using kits provided by Beijing Solarbio Science & Technology Co., Ltd., Beijing, China. A change of 0.001 in the absorbance of the reaction solution at a certain wavelength per minute was defined as one enzyme activity unit, which was expressed as U·mg^−1^ protein. The changes in absorbance of the reaction solution at 420, 234, 450, 470, 450, 240, and 290 nm per minute were used to calculate PPO, LOX, PLD, POD, SOD, CAT, and APX activity, respectively. PAL activity was calculated by measuring the increasing rate of absorption at 290 nm. A change of 0.1 in the absorbance at 290 nm per minute for each gram of tissue in the reaction system per mL was defined as an enzyme activity unit for PAL, expressed as U·mg^−1^ protein.

### 2.7. Determination of Antioxidant Capacity (DPPH, ABTS, and FRAP)

Then, 2,2-diphenyl-1-picrylhydrazyl (DPPH) scavenging activity was determined based on the method reported by Proestos et al. [17] with some modifications. Briefly, 2800 μL DPPH solution (60 μM) and 200 μL of the supernatant (sample extract) or 70% ethanol (control) was added to a test tube and incubated in the dark for 30 min. The ultraviolet absorbance of the mixture at 517 nm was determined and recorded as A. The DPPH radical scavenging activity of each sample was calculated as the percent inhibition according to the following equation:

DPPH radical scavenging activity of the sample (%) = [(A_control_ − A_sample_)/A_control_] × 100%,

where A_control_ is the absorbance of the eggplant sample replaced by 70% ethanol, and A_sample_ is the absorbance of the eggplant sample solution. The 2,2′-azinobis-(3-ethylbenzothiazoline-6-sulfonic acid) (ABTS) scavenging capacity was measured using the method of Proestos et al. [17]. Briefly, ABTS working reagent was prepared by mixing 7 mM ABTS solution with 4.9 mM K_2_S_2_O_8_ solution (volume ratio 1:1) and incubating it in the dark for 24–48 h. The working reagent was diluted with methanol before use until the absorbance in the samples reached 0.7 at 734 nm. Thereafter, eggplant sample extract (0.5 mL) was added to 3.5 mL of ABTS working reagent, and the mixture was kept in the dark for 60 min at a standard temperature of 30 °C. The absorbance was measured at 734 nm. The ABTS radical scavenging activity of each sample was calculated as the percent inhibition according to the following formula:

ABTS inhibition (%) = [A_0_ − (A_1_ − A_2_)/A_0_] × 100%,

where A_0_ is the absorbance of the eggplant sample replaced by distilled water, A_1_ is the absorbance of the eggplant sample solution, and A_2_ is the absorbance of the ABTS working reagent replaced by distilled water. The Benzie and Strain method [18] was used to evaluate the ferric-reducing antioxidant power (FRAP) of fresh-cut eggplant samples. The FRAP reagent was prepared as follows: 300 mmol/L acetic acid buffer (pH 3.6), 10 mmol/L 2,4,6-tri(2-pyridyl)-s-triazine (TPTZ) (dissolved in 40 mmol/L HCl), and a mixture of 20 mmol/L FeCl_3_ solution (volume ratio 10: 1:1). The reagent was then mixed with 100 L of supernatant in 3.6 mL increments (eggplant sample). After 30 min of incubation at 37 °C, the absorbance of the mixtures was measured at 593 nm. Using the FeSO_4_ calibration curve, FRAP results were expressed as mol Fe^2+^ equivalents per gram of extract.

### 2.8. Determination of Total Phenol (TP) Content

The Folin–Ciocalteu method [19] was used to determine total phenol content, and kits (Suzhou Grace Biotechnology Co., Ltd., Suzhou, China) were used to determine TP content. The absorbance at 760 nm was measured and used to calculate TP.

### 2.9. Statistical Analyses

The experimental data were plotted using Excel, and significant differences were determined by ANOVA and Duncan’s multiple range test of SPSS 22.0.

## 3. Results

### 3.1. Phenotypic Characteristics of Physiological Responses of Fresh-Cut Eggplant

**Figure 1:**
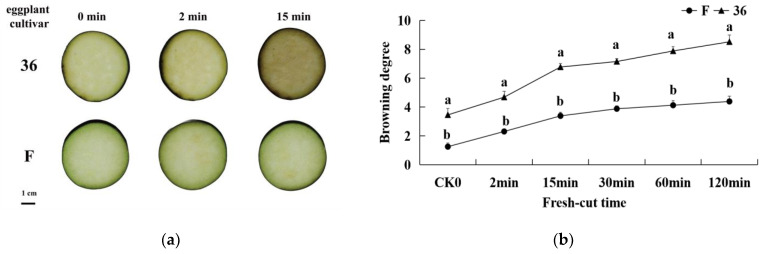
Browning phenotype of slices (**a**) and browning degree (**b**) of browning-sensitive (‘36′) and browning-resistant (‘F’) cultivars after cutting.

The browning-sensitive (‘36′) and browning-resistant (‘F’) cultivar fruit samples showed different degrees of browning within the treatment time (Figure 1a). The surface color of the two original eggplant materials was bright white and green. However, as the fresh-cut time was prolonged, the surface color of the slices showed different degrees of browning. As shown in Figure 1a, the flesh of ‘36′ began turning brown 2 min after cutting and became prominently brown 15 min after cutting. In contrast, the material ‘F’ did not show prominent browning until 15 min after cutting.

After the cutting process of fruits and vegetables, due to enzymatic browning at the cutting site induced by mechanical injury, BD is used as an indicator that reflects the degree of browning of fresh-cut fruits and vegetables. The changes in BD in eggplant slices with time after cutting are shown in Figure 1b. With the extension of time after cutting, the BD of the samples of the two types of eggplant increased gradually, but the BD of ‘36′ was significantly higher than that of ‘F’ (*p* < 0.05).

### 3.2. Qualitative and Quantitative Analysis of Metabolites

Based on VIP > 1 for the first principal component in the OPLS-DA, and *p*-value < 0.05, we identified the differential metabolites among the nine contrasting groups (Table S1). In total, 8475 metabolites were identified, among which 686 (398 up-regulated, 288 down-regulated), 752 (392 up-regulated, 360 down-regulated), and 851 (411 up-regulated, 440 down-regulated) metabolites showed differential accumulation in the F 15 min/36 15 min, F 2 min/36 2 min, and F CK0/36 CK0, respectively. Furthermore, 754 (333 up-regulated, 421 down-regulated) differential metabolites were detected in the F 2 min/F CK0 comparison group, 653 (332 up-regulated, 321 down-regulated) in the F 15 min/F CK0 comparison group, and 625 (429 up-regulated, 196 down-regulated) in the F 15 min/F 2-min comparison group. A total of 520 (324 up-regulated, 196 down-regulated) differential metabolites were detected in the 36 2 min/36 CK0 comparison group, 468 (234 up-regulated, 234 down-regulated) in the 36 15 min/36 CK0 comparison group, and 658 (255 up-regulated, 403 down-regulated) in the 36 15 min/36 2 min comparison group.

### 3.3. Differential Metabolites (DEMs) Screening, Functional Annotation, and Enrichment Analysis

The expression levels of the differential metabolites in the F CK0/36 CK0 comparison group are shown in Figure S1a. The results showed that 369 lipids and lipid-like metabolites had significant changes. Specifically, 9(S)-HODE, PC (19:0/12:0) and stearidonic acid were significantly increased, whereas 9,10-DiHOME and glycerophosphocholine were significantly decreased; 63 flavonoids and isoflavonoids (including ferulyl and catechin 3-O-rutinoside) were significantly increased, whereas quercetin, delphinidin, and luteolin were significantly decreased; eight phenolic acids including cinnatriacetin A were significantly increased, while quinic acid and chlorogenic acid were significantly decreased; 75 organic acids (including histidinyl-cysteine and L-aspartic acid) were significantly increased, while L-tyrosine, L-asparagine, and alanyl-phenylalanine were significantly decreased. Additionally, 21 benzenoids including zingerone and diphenoxylate were significantly increased, and gingerol was significantly decreased. Significant differences were found in 434 lipids and lipid-like molecules, 32 flavonoids and Isoflavonoids, 58 organic acids, 27 benzenoids in F 2 min/36 2 min (Figure S1b).

The expression levels of the differential metabolites in the F 15 min/36 15 min comparison group are shown in Figure S1c. The results showed that 400 lipids and lipid-like molecules had significant changes. Moreover, 9(S)-HODE, PS(P-20:0/21:0), PA(20:0/14:0), and PC(16:0/22:0) were significantly increased, and PS(22:0/20:2(11Z,14Z)) and PG(22:2(13Z,16Z)/0:0) were significantly decreased; 21 flavonoids and isoflavonoids including pyranocyanin B and ferulylglucoside were significantly increased, while kaempferol and daidzin were significantly decreased. Two phenolic acids (2-hydroxycinnamic acid and feruloyl C1-glucuronide) were significantly decreased); 41 organic acids (including oxidized glutathione and s-nitrosoglutathione) were significantly increased, while L-valine and L-asparagine were significantly decreased; 14 benzenoid had significant changes. The differential metabolite heat maps of the other groups are shown in Figure S1.

### 3.4. Enrichment Analysis of DEMs

**Figure 2:**
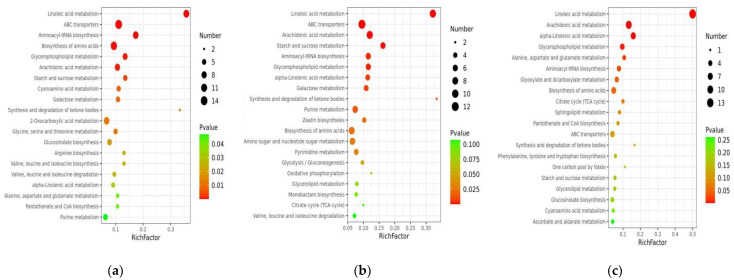
KEGG analysis of the DEMs in (**a**) F CK0/36 CK0; (**b**) F 2 min/36 2 min; and (**c**) F 15 min/36 15 min. Note: The ordinate refers to metabolic pathway; abscissa is the enrichment factor (Enrichment factor = the number of metabolites with significant difference/the number of total metabolites in the pathway). The color change from red to green means that the *p*-value increases successively.

The top 20 metabolic pathways with significant enrichment were selected for bubble mapping to better comprehend the differential metabolic pathways of numerous different samples by comparing the differential metabolites in the KEGG database (Figure 2). In the F CK0/36 CK0 comparison group, linoleic acid metabolism, amino acid biosynthesis, and arachidonic acid metabolism were significantly enriched (Figure 2a). In the F 2 min/36 2 min comparison group, linoleic acid metabolism, arachidonic acid metabolism, and starch and sucrose metabolism were significantly enriched (Figure 2b). Linoleic acid metabolism, arachidonic acid metabolism, alpha-linolenic acid metabolism, aspartate, and glutamate metabolism were significantly enriched in the F 15 min/36 15 min comparison group (Figure 2c). The differential metabolite bubble maps of the other comparison groups are shown in Figure S2. From the perspective of differential metabolites, lipid metabolism was enhanced, unsaturated fatty acids such as linoleic acid and linolenic acid were oxidized to generate lipid hydroperoxides, membrane lipid peroxidation products accumulated, cell membrane structure was damaged, and phenolic substrates were oxidized by polyphenol oxidase to produce browning.

### 3.5. Integrated Analysis of the Transcriptome and Metabolome of the Fresh-Cut Eggplant

Combined with the reported transcriptome data of (SRA PRJNA679701), we analyzed the relationship between genes and metabolites that may be involved in the browning of fresh-cut eggplant.

#### 3.5.1. Changes in Membrane Lipid Composition of Fresh-Cut Eggplant

**Figure 3:**
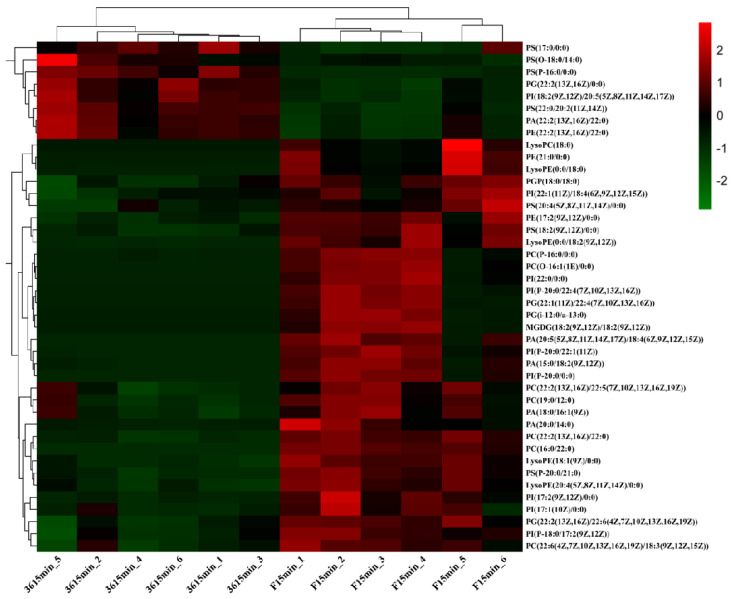
Heat map of membrane lipid content difference of fresh-cut eggplant in F 15 min/36 15 min. Note: Hierarchical clustering of DEMs (*p* < 0.05 and VIP > 1 were considered as DEMs between two groups). The rows in the heat map represent metabolites, and the columns represent different samples. Up-regulated metabolites are indicated in red and down-regulated metabolites in green.

The change in membrane lipid composition due to environmental stress determines the changes in membrane permeability and fluidity. We compared the lipids content of ‘36′, which was significantly browned 15 min after cutting, with ‘F’, which was not. As shown in Figure 3, there were three types of glycerolipids (TG, DG, and MGDG), six types of glycerophospholipids (PA, PC, PE, PG, PI, and PS), and two types of lysophosphatides (LPC and LPE). The results showed that almost all differentially accumulated glycerolipids and glycerophospholipids had higher levels in the ‘F’ fruit relative to the ‘36′ fruit; specifically, PC, PI, and PG displayed a highly significant difference in accumulation. These results showed that, compared with the eggplant material ‘F’ (browning-resistant), the lipid components of ‘36′ (browning-sensitive) is lower, or the lipid degradation is rapid after cutting, and the cell membrane destruction is more prominent, which may be an important reason for the serious browning.

**Figure 4:**
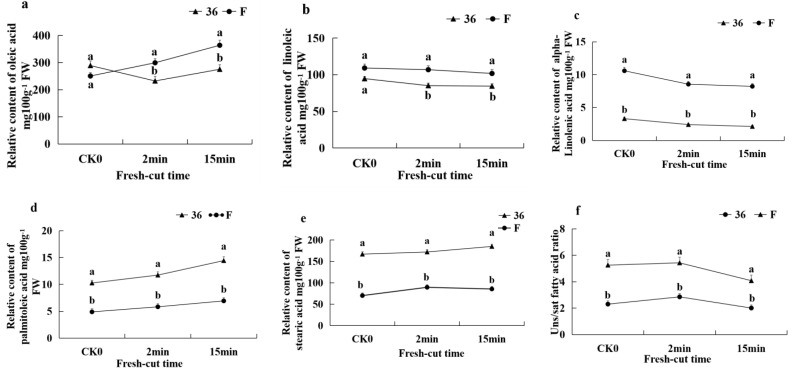
Changes in fresh-cut eggplant fatty acid composition and content. (**a**) Oleic acid relative content; (**b**) linoleic acid relative content; (**c**) linolenic acid relative content; (**d**) palmitic acid relative content; (**e**) stearic acid relative content; and (**f**) ratio of unsaturated to saturated fatty acids, U/S. Note: The means ± SE of six replicates are shown. Different letters above the bars indicate the statistically significant difference at *p* < 0.05.

Five major fatty acids were identified, comprising three unsaturated fatty acids (USFAs), oleic acid, linoleic acid, linolenic acid, and two saturated fatty acids (SFAs), palmitic acid and stearic acid. Between ‘36′ and ‘F’, there were significant differences in the relative content of the three types of USFAs and two types of SFAs. The relative content of USFAs in ‘F’ fruit was significantly higher than in ‘36′ fruit. (Figure 4a–c). However, in contrast to the USFAs, the relative content of SFAs in ‘F’ fruit was significantly lower than that in ‘36′ fruit (Figure 4d,e). Similarly, the ratio of unsaturated to saturated fatty acids (U/S) were significantly higher in ‘F’ than those in samples for ‘36′ fruit (Figure 4f).

#### 3.5.2. Expression of DEGs and DMEs Related to Lipid Metabolism

**Figure 5:**
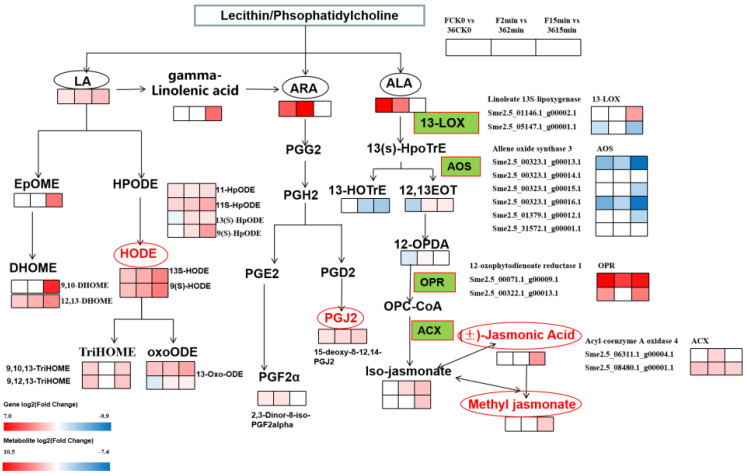
The DEGs and DEMs involved in LA, AA, and ALA metabolism in response to browning. The green pattern represented the genes that changed after cutting. Note: The rectangle was divided into three equal parts representing DEGs or DEMs in F CK0/36 CK0, F 2 min/36 2 min, and F 15 min/36 15 min, respectively. Red represents up-regulation and blue represents down-regulation.

To further clarify the role of lipid metabolism in the browning process of fresh-cut eggplants, we analyzed the changes in differential genes (DEGs) and differential metabolites (DMEs) in lipid metabolism. According to the KEGG enrichment results, DMEs are mainly enriched in unsaturated fatty acid metabolism, including linoleic acid (LA), arachidonic acid (AA), and alpha-linolenic acid (ALA). Therefore, we focused on the analysis of DEGs and DMEs involved in these metabolic processes (Figure 5). The metabolism of PUFA substrates (LA, AA, and ALA) occurs after phospholipase (PL) hydrolyzes phosphatidylcholine in cell membranes catalyzed by members of the PL family, mainly PLA2. For the LA metabolism processes, one EpoME (9,10-Epoxyoctadecenoic acid) and two DHOMEs (9,10-DHOME and 12,13-DiHOME), catalyzed by CYP enzymes, were significantly upregulated in ‘F’ vs. ‘36′. In contrast, 10 DMEs (11-HpODE, 11S-HpODE, 13(S)-HpODE, 9(S)-HpODE, 13S-HODE, 9(S)-HODE, 9,10,13-TriHOME, 9,12,13-TriHOME, 13-Oxo-ODE, and 13-OxoODE) catalyzed by LOX enzymes were identified, all of which were upregulated in ‘F’ vs. ‘36′. In addition, five 9-LOX-encoding genes and two 13-LOX-encoding genes were significantly different in ‘F’ vs. ‘36′ comparison groups. Linoleyl-CoA desaturase catalyzes LA to produce linolenic acid, which then produces arachidonic acid, which enters the AA metabolism. Two DMEs (15-deoxy-δ-12,14-PGJ2 and 2,3-Dinor-8-iso-PGF2alpha), which are powerful antioxidants, were significantly upregulated in ‘F’ vs. ‘36′. Additionally, 9(s)-HpoTrE and 13(s)-HpoTrE were associated with catalysis by 9-LOX and 13-LOX, respectively. With the change in related enzyme genes, related metabolites (13(S)-HOTrE, 12,13S-EOT, 12-OPDA, (3R,7S)-iso-jasmonic acid, (±)-jasmonic acid, and methyl jasmonate) accumulated. There were six AOS that were downregulated in ‘F’ vs. ‘36′, while three ADH, two OPR, and two ACX were upregulated. The results showed that the differential genes and metabolites related to unsaturated fatty acid metabolism in fresh-cut eggplant were taken conjointly to respond to oxidative stress.

#### 3.5.3. Expression of DEGs and DMEs Related to Phenylpropane Biosynthesis

**Figure 6:**
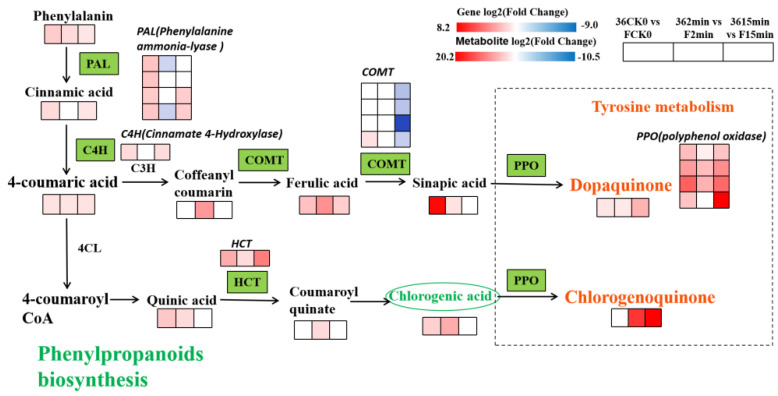
The DEGs and DEMs of Phenylpropanoids biosynthesis during fresh-cut. Note: The green pattern represented the genes that changed after cutting. The rectangle was divided into three equal parts representing DEGs or DEMs in 36 CK0/F CK0, 36 2 min/F 2 min, and 36 15 min/F 15 min, respectively. Red represents up-regulation and blue represents down-regulation.

Several phenylpropane substances have been identified in differential metabolites; hence, we performed a co-expression analysis of differential genes and metabolites of the phenylpropane biosynthetic pathway (Figure 6). The results showed that compared with the eggplant material ‘F’ (browning-resistant), ‘36′ (browning-sensitive) contains relatively rich phenolic acids after cutting. Phenylalanine produces cinnamic acid under the action of PAL, then 4-coumarinic acid under the action of cinnamic acid-4-hydroxylase (C4H), which in turn produces phenolic acids such as ferulic acid, mustardic acid, quinine acid, and chlorogenic acid. These phenolic acids showed substantial upregulation in the three comparison groups of ‘36′ vs. ‘F’. Subsequently, they were oxidized by PPO through the tyrosine metabolic pathway to form quinones such as dopamine quinone and chlorogenic quinone. Compared with F, dopaquinone and chlorogenic quinone were significantly upregulated in ‘36′ after cutting for 2 min and 15 min. The more pronounced browning in ‘36′ is attributed to the accumulation of quinones.

#### 3.5.4. Expression of DEGs and DMEs Related to Glutathione Metabolism

**Figure 7:**
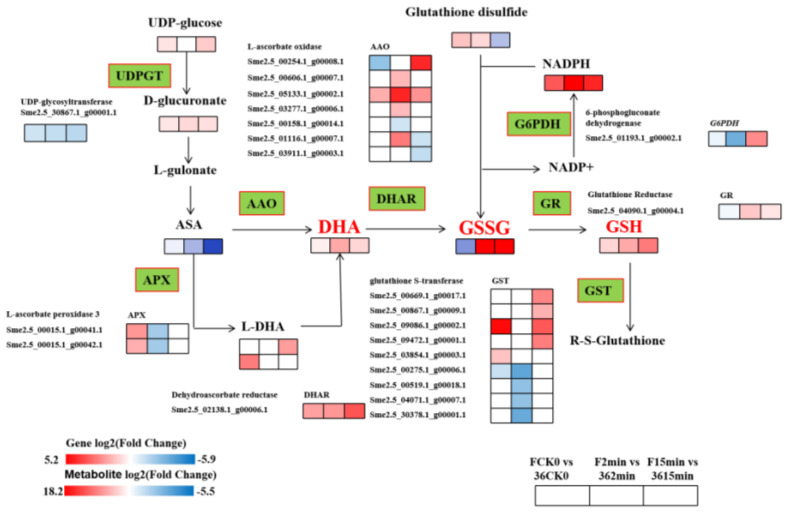
The DEGs and DEMs of ASA–GSH metabolism during fresh-cut eggplant browning. Note: The green pattern represented the genes that changed after cutting. The rectangle was divided into three equal parts representing DEGs or DEMs in F CK0/36 CK0, F 2 min/36 2 min, and F 15 min/36 15 min, respectively. Red represents up-regulation, and blue represents down-regulation.

To examine the effects of fresh-cut eggplants on genes and metabolites related to glutathione metabolism, the interaction of differential genes and metabolites was analyzed (Figure 7). Ascorbic acid (ASA) generates dehydrogenated ascorbic acid (DHA) under the action of AAO, and then oxidized glutathione (GSSG) under the action of dehydroascorbic acid reductase (DHAR), and finally reduced to glutathione (GSH) by glutathione reductase (GR). Among them, the expression of 7 *AAO*, 2 *APX*, 1 *DHAR*, 1 *GR,* and 9 *GST* genes showed significant differences between the two materials, and the metabolically generated GSSH and GSH were significantly upregulated in the ‘F’ vs. ‘36′ comparison group, indicating that after ‘F’ fruit was freshly cut, the antioxidant substance glutathione accumulated, which improved its antioxidant capacity and in turn made it less susceptible to browning.

#### 3.5.5. Malondialdehyde Content, H_2_O_2_ Content, and O_2_^−^ Production Rate

**Figure 8:**
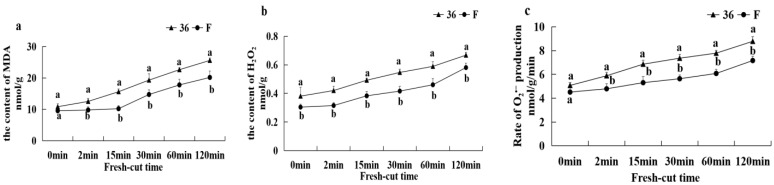
The MDA content (**a**), H_2_O_2_ content (**b**), and O_2_^−^ production rate (**c**) of eggplant fruit after cutting.

As shown in Figure 8, the MDA content of both eggplant materials gradually increased with increasing fresh-cutting time after the fresh-cut treatment, while the H_2_O_2_ content and O_2_^−^ production rate both showed a rapid increase. In addition, the MDA content, H_2_O_2_ content and O_2_^−^ production rate in ‘36′ were significantly higher than those in ‘F’ (*p* < 0.05) after the fresh-cut treatment.

#### 3.5.6. Activities of PPO, LOX, PLD, POD, PAL, and TP Content

**Figure 9:**
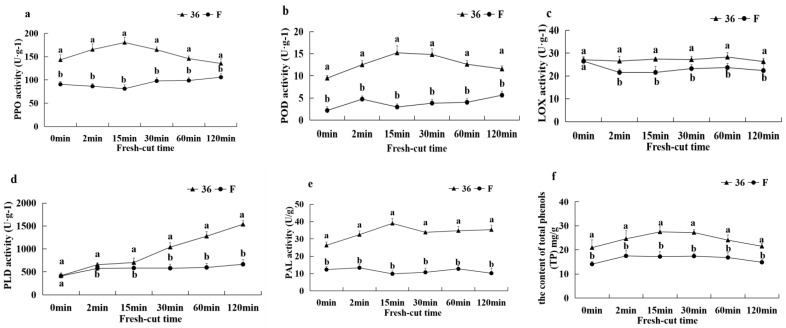
The PPO activity (**a**), POD activity (**b**), LOX activity (**c**), PLD activity (**d**), PAL activity (**e**), and TP content (**f**) of eggplant fruit after cutting.

As shown in Figure 9, the PPO, POD, PAL activities and TP content of ‘36′ fruit were significantly higher than those of ‘F’ fruit after 0 min of fresh-cut, while there were no significant differences in LOX and PLD activities between the two materials. The PPO, POD, and PAL activities and TP content of ‘36′ fruits showed an increasing and then decreasing trends with the extension of fresh-cut time, while LOX activity showed no significant change and PLD activity increased significantly after 15 min. The PPO activity of ‘F’ fruit showed an increasing trend after 30 min of fresh-cutting, while all other indexes did not change significantly during the fresh-cutting process. Throughout the browning process of fresh-cut fruits, the PPO, POD, LOX, PLD, and PAL activities and TP contents of ‘36′ fruits were significantly higher than those of ‘F’, and the changes of PAL activities and TP contents showed a consistent trend, and high PAL activities were accompanied by high TP contents. These results suggest that higher PPO, LOX, PLD, POD, and PAL activities and TP content were correlated with higher browning.

#### 3.5.7. Antioxidant Capacity and the Activities of CAT, APX, and SOD

**Figure 10:**
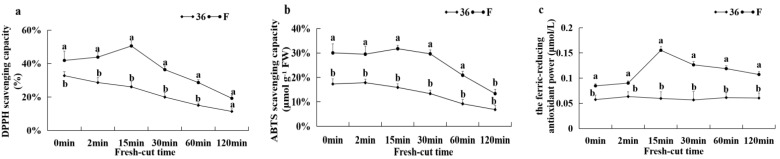

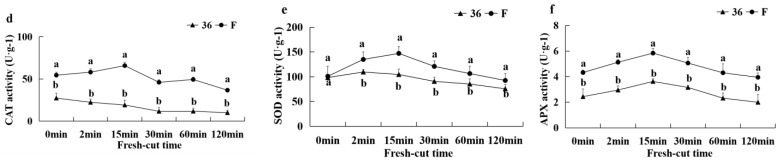
DPPH radical scavenging ability (**a**), ABTS radical scavenging activity (**b**), FRAP scavenging ability (**c**), CAT activity (**d**), SOD activity (**e**), and APX activity (**f**) of eggplant fruit after cutting.

Figure 10 shows the antioxidant capacity and antioxidant enzyme activities of CAT, APX, and SOD after cutting the eggplant. The DPPH radical scavenging ability, ABTS radical scavenging ability, and the scavenging ability of FRAP of eggplant material ‘F’ showed a trend of increasing and then decreasing after cutting, while the DPPH radical scavenging ability and ABTS radical scavenging ability of eggplant material ‘36′ showed a decreasing trend after cutting and no significant change in the scavenging ability of FRAP. The overall antioxidant capacity of ‘F’ was significantly higher than that of ‘36′ fruits throughout the browning process of fresh-cut fruits (Figure 10a–c). After cutting, the APX and SOD activities of both eggplant fruits showed a tendency to increase and then decrease, with the SOD activity of ‘F’ reaching a maximum at 15 min and the APX activity of ‘36′ fruit reaching a maximum at 2 min in both cases. The change in CAT activity of ‘F’ fruit with increasing fresh-cutting time was consistent with APX, while CAT activity of ‘36′ fruit decreased slowly. The CAT, SOD, and APX activities of ‘F’ fruit were significantly higher than those of ‘36′ fruit after cutting (Figure 10d–f), indicating that increasing antioxidant enzyme activities helped to ameliorate browning of fresh-cut eggplant.

## 4. Discussion

Fruit browning includes enzymatic browning and non-enzymatic browning enzymatic browning and is considered to be the main cause of fresh-cut fruit and vegetable browning caused by the catalytic effect of phenol oxidase on phenolic substrates [6,11]. The oxidation of phenolic substances by polyphenol oxidase (PPO) and peroxidase (POD) is a key factor affecting the browning of fruits [20,21]. In the presence of H_2_O_2_, different kinds of phenolic compounds can be oxidized by PPO and POD, causing enzymatic browning of postharvest fruits and vegetables, such as potato [22]. Both HPLC and LC-MS analyses of the eggplant fruits revealed that, in all eggplant genotypes, chlorogenic acid was the main phenolic compound, with double the amount present in the highly-browning eggplant AM199 relative to the lower-browning AM086 [14]. Our results showed that mechanical damage can induce the activity of the phenylpropane metabolic enzyme system, of which PAL is the first key enzyme of the system. Cleavage-induced mechanical damage significantly activates the gene expression of phenylalaninelyase PAL; improves PAL activity [23,24]; induces the accumulation of phenolic acids such as coumic acid, ferulic acid, mustardic acid, quinic acid, and chlorogenic acid; significantly upregulates the expression of PPO genes; and elevates polyphenol oxidase activity, which oxidizes phenolic acids to quinones. The generation of dopaquinone and chlorogenic quinone induces the occurrence of browning; we found that, compared with dopaquinone, chlorogenic quinone has a higher content in ‘36′, and observed a significant difference in the starting chlorogenic acid content of the two eggplant materials where the content of chlorogenic acid in 36 CK0 was significantly higher than F CK0. This suggests that chlorogenic acid may be the main browning substrate of fresh-cut eggplant. Docimo et al. [14] reported a positive relationship between chlorogenic acid and total phenols in the genetic lines studied, but no correlation between flesh browning and high chlorogenic acid content or high PPO activity. This contradicts our results, which is possibly attributed to the different substrate specificity and distinct substrate affinity of different eggplant cultivars for PPO.

ROS are important mediators of plant stress responses [25]. When cells undergo stress, the ability of the fruit to scour free radicals is reduced, leading to the accumulation of ROS, thereby accelerating membrane lipid peroxidation, damaging cell structure, improving membrane permeability, and reducing its antioxidant capacity [25]. Studies on carrot [25] and pitaya [3] observed that a large amount of ROS is produced by physical damage. You et al. [26] also confirmed that the H_2_O_2_ content of lotus seeds increased continuously after cutting. The DPPH, ABTS+, and ferric-reducing antioxidant power (FRAP) assay is a simple and accurate method to evaluate the free radical scavenging activity of fruit and vegetable extracts [27]. A previous study has suggested that the lower the temperature, the stronger the antioxidant capacity of fresh-cut apples, and the lower the browning degree [28]. This is supported by our results, since after the cell membrane of fresh eggplant was damaged after cutting, ROS (H_2_O_2_, O_2_^−^) and oxidation product of membrane lipid (MDA) contents increased with time. ROS and oxidation product levels of ‘36′ were significantly higher than those of ‘F’, which reduced its antioxidant capacity and increased the cell membrane permeability, causing intense fruit browning.

The ASA–GSH cycle is involved in the elimination of ROS in plants. Plants under stress can remove excess ROS through reactive oxygen scavenging enzymes (CAT, SOD, and APX) and non-enzymatic antioxidants (including ascorbic acid (AsA) and glutathione (GSH)) [29]. AsA and GSH are two important antioxidant substances in the AsA–GSH cycle pathway, which plays an important role in scavenging ROS in plants [30]. In this cycle, APX uses AsA as a reaction substrate to reduce H_2_O_2_ to H_2_O; AsA can be oxidized to monodehydroascorbic acid and dehydroascorbic acid. GSH participates in the regeneration of AsA-reducing agents when fully oxidized DHA is produced with GSH being oxidized concurrently. The oxidized glutathione (GSSG) is reduced to GSH by glutathione reductase (GR) [31]. GST is an enzyme that uses glutathione to participate in plant defense metabolism and reduce secondary harmful products of stress-induced ROS production. Sun et al. [11] discovered that FA could decrease the secondary injury products of ROS under drought stress by expressing GST. In this study, we found that *GST* genes were upregulated in the F 15 min/36 15 min comparison group. The antioxidant substances GSSH and GSH in ‘F’ fruit were significantly upregulated by a series of antioxidant enzymes (APX, GST, and AAO) compared with ‘36′. However, the ASA content showed the opposite result, i.e., down-regulated expression in ‘F’, possibly since through the ASA–GSH cycle, ASA is consumed as a substrate to generate large amounts of GSH. Therefore, we speculated that compared with ‘36′ fruits, ‘F’ fruits have higher antioxidant enzyme (APX, GST, AAO, SOD, CAT, and CAT) activity, and under the regulation of the corresponding enzyme genes, a large amount of antioxidant substance-glutathione was generated, which explains the strong antioxidant capacity and resistance to browning.

The occurrence of enzyme-induced browning of fruits is closely related to the integrity of the cell membrane [11]. By maintaining cell mobility and permeability, membrane lipids serve a vital role in supporting proper physiological metabolism [32]. Membrane lipid metabolism is dependent on phospholipid hydrolysis and changes in fatty acid content, according to previous research [33]. As the major component of membrane lipids, phospholipids can play a role in a variety of physiological processes via signaling chemicals produced by metabolism [7]. Previous research has found that after treating ‘Nanguo’ pears with n-butanol, the degradation of membrane lipids in the core tissue was reduced, while the total membrane lipid content, MGDG content, and PA, PC, PE, PI, PS, LPC, and LPG content were higher than the control [11]. Similarly, three types of glycerol (TG, DG, and MGDG), six types of glycerophospholipids (PA, PC, PE, PG, PI, and PS), and two kinds of lysophospholipids (LPC and LPE) were identified in our differential metabolites, and their levels were significantly (*p* < 0.05) higher in ‘F’ than in ‘36′. In particular, the difference in the accumulation of PC, PI, and PG was highly significant. U/S is an important factor that reflects the degree of cell membrane unsaturation [34]. Reduced U/S can cause cell membrane compartmentalization to break down, allowing phenolic enzymes to come into contact with phenolic substrates and catalyze phenol oxidation, resulting in browning products [35]. A greater U/S value was associated with stronger stress tolerance and lower BI in pears, according to Sun et al. [11]. Our findings revealed that following cutting, cell membrane permeability increased momentarily, accompanied by lower U/S values. After the cutting procedure, increased SFA levels, decreased USFAs in the cell membrane, and membrane phospholipid breakdown induced more serious browning in the ‘36′ fruit. PLD, lipase, and LOX are essential enzymes in membrane lipid metabolism that may cause phospholipid breakdown, according to a previous study [35]. In our study, after cutting, the LOX and PLD activity of eggplant fruit was significantly increased, while the PLD, lipase, and LOX coding genes were significantly different, corroborating the results of Lin et al. [35], which suggests that the degradation of cell membrane lipids under phospholipase catalysis accelerated the occurrence of browning.

During oxidative stress, polyunsaturated fatty acids (PUFAs) linked with membrane phospholipids are primary targets of ROS alteration [36]. PUFAs are highly reactive molecules that can be rapidly transformed into substances with physiological activity, namely oxylipids, which play an important role in plant growth, development, and plant responses to environmental stress [37]. Omega 6 PUFAs such arachidonic acid (ARA) and linoleic acid (LA), as well as omega 3 PUFAs such as linolenic acid (ALA), eicosapentaenoic acid (EPA), and docosahexaenoic acid (DHA), are common oxylipid synthesis substrates [38]. After phospholipase hydrolyzes phospholipids in cell membranes, which is catalyzed by members of the phospholipase (PL) family, primarily PLA2, PUFA substrates are metabolized [36]. ALA is catalyzed to form 13-Hydroperoxylinolenic acid (13-HPOT) from 13-LOX and metabolized to jasmonic acid (JA) by allene oxide synthase (AOS). JA leaves the peroxisome as a signal molecule and is methylated by jasmonic acid carboxyl methyltransferase (JMT) in the cytoplasm to form methyl jasmonate (MEJA) [39]. The JA signal transduction pathway has been clarified [39]. The level of JA in normal plant cells is low and increases sharply when plants are exposed to environmental stress. The expression of the JA responsive gene is activated to cope with stress injury [39]. Plants protect against further damage by inducing antioxidant defense enzymes in response to a variety of environmental stresses (biotic and abiotic) [40]. Exogenous MeJA treatments can induce the activities of antioxidant enzymes (SOD, APX, CAT, and POD) and gene expression in postharvest produces during storage [41,42]. The results showed that MEJA treatment reduced the browning index, loss of membrane integrity, and enzymatic browning of tissues near the pineapple core and increased antioxidant activity and AsA concentration [43]. Furthermore, the precursor of JA synthesis, 12-oxygen-phytodienic acid (OPDA), has been reported to trigger a specific set of genes in Arabidopsis as an intermediate molecule of known signaling function [44]. LA can be metabolized through the LOX pathway to produce hydroxylperoxadienoic acid (HpODE). These intermediate metabolites are lipid hydroperoxides, which can induce to oxidative stress. Unstable HpODE can be further metabolized into the corresponding hydroxyl derivatives (HODE) and keto-based derivatives (oxoODE) [45]. Some oxylipids produced with ARA substrates, such as 15-DPGJ2, can lower the formation of active metabolites from sources, either directly or indirectly, such as the mitochondria [46]; 15-DPGJ2 can promote the production of antioxidant enzymes and antioxidants by activating the expression of antioxidant genes such as heme oxygenase-1 (HO-1), SOD, GSH-Px, and ferritin, thereby reducing oxidative stress [47]. In the metabolome data of the ‘F’ and ‘36′ fresh-cut fruits, it was found that differential metabolites were mainly enriched in unsaturated fatty acid metabolism, such as linoleic acid, linolenic acid, and arachidonic acid. Therefore, we focused on the analysis of the differential genes and metabolites involved in this metabolism and showed that with the differential expression changes of the corresponding genes in the resistant fruits, the oxylipid content of most plants, such as JA, MEJA, OPDA, HODE, and 15-DPGJ2, was significantly increased. Reactive oxygen species were eliminated by improving antioxidant enzyme activity and gene expression. These results suggest that plant oxylipids play a major role during the early signal transmission of fresh-cut stress in eggplant and in the tolerance mechanism of the browning-tolerant variety.

## 5. Conclusions

The mechanism of browning in fresh-cut eggplant fruit was investigated. The loss of membrane integrity and increase in membrane lipid degrading enzymes’ activity and gene expression, such as LOX, PLD, and lipase, may have accelerated the degradation of membrane lipids and membrane USFAs, resulting in fresh-cut eggplant browning. However, the fresh-cut treatment resulted in PAL and PPO activity increased, phenolic acid accumulation, and facilitated the interaction of PPO and phenolic acid. The generation of dopaquinone and chlorogenic quinone induced the occurrence of browning. Simultaneously, most oxylipids accumulated in plants can eliminate ROS and inhibit browning by increasing the activity of antioxidant enzymes (SOD, CAT, and APX) and activating the ASA–GSH cycle, suggesting that plant oxylipids play a role during the early signal transmission of cutting stress in eggplant and participate in the tolerance mechanism of the browning-tolerant variety. These results provide insights into the molecular mechanisms underlying fresh-cut eggplant browning. However, further studies are warranted to assess the enzymatic nature of genes directly responsible for browning.

## Data Availability

The transcriptome datasets supporting the conclusions of this article are available in the National Center for Biotechnology Information (https://www.ncbi.nlm.nih.gov/sra/PRJNA679701, accessed on 20 November 2020). The data charts supporting the results and conclusions are included in the article and additional files.

## Supplementary Materials

The following supporting information can be downloaded at https://www.mdpi.com/article/10.3390/foods11081174/s1, Figure S1: The differential metabolite heat maps of comparison groups. (a) F CK0/36 CK0 group; (b) F 2 min/36 2 min group; (c) F 15 min/36 15 min group; (d) F 15 min/F CK0 group; (e) F 15 min/F 2 min group; (f) F 2 min/F CK0 group; (g) 36 15 min/36 CK0; (h) 36 15 min/36 2 min; (i) 36 2 min/36 CK0; Figure S2: The differential metabolite bubble maps of comparison groups. (a) F 15 min/F CK0 group; (b) F 15 min/F 2 min group. (c) F 2 min/F CK0 group; (d) 36 15 min/36 CK0; (e) 36 15 min/36 2 min; (f) 36 2 min/36 CK0; Table S1: Differential metabolites among the nine contrastive groups.
