# Supplementary material for: Metabolomic Analysis, Combined with Enzymatic and Transcriptome Assays, to Reveal the Browning Resistance Mechanism of Fresh-Cut Eggplant
Source: Foods. 2022 Apr 18;11(8):1174. doi: 10.3390/foods11081174 (PMC9031582; doi:10.3390/foods11081174)
Supplement: Supplementary file 1 [file foods-11-01174-s001.zip › Figures SM.pdf]

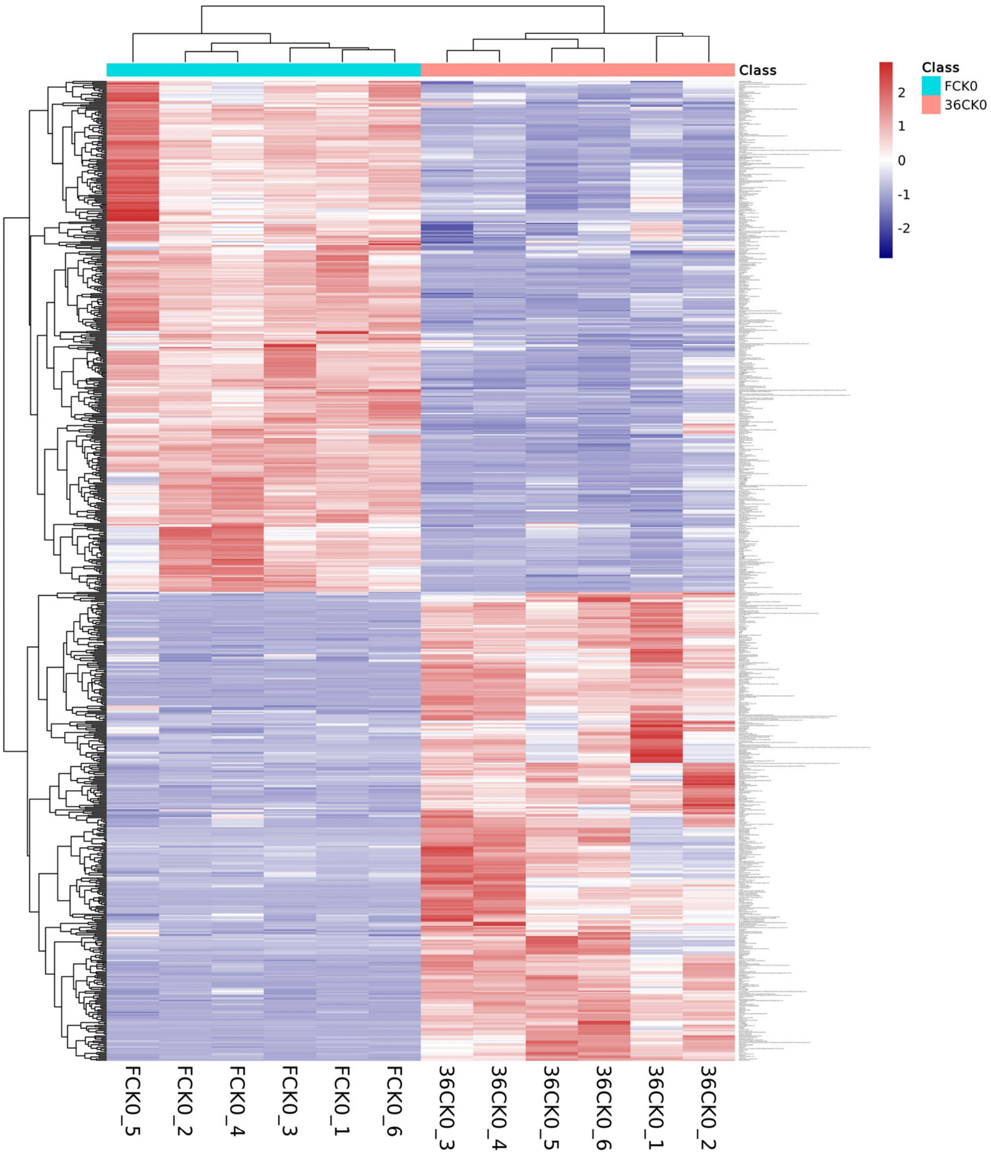
Class
FCK0
36CK0
2
1
0
-1
-2
Class
FCK0_5
FCK0_2
FCK0_4
FCK0_3
FCK0_1
FCK0_6
36CK0_3
36CK0_4
36CK0_5
36CK0_6
36CK0_1
36CK0_2

(a)

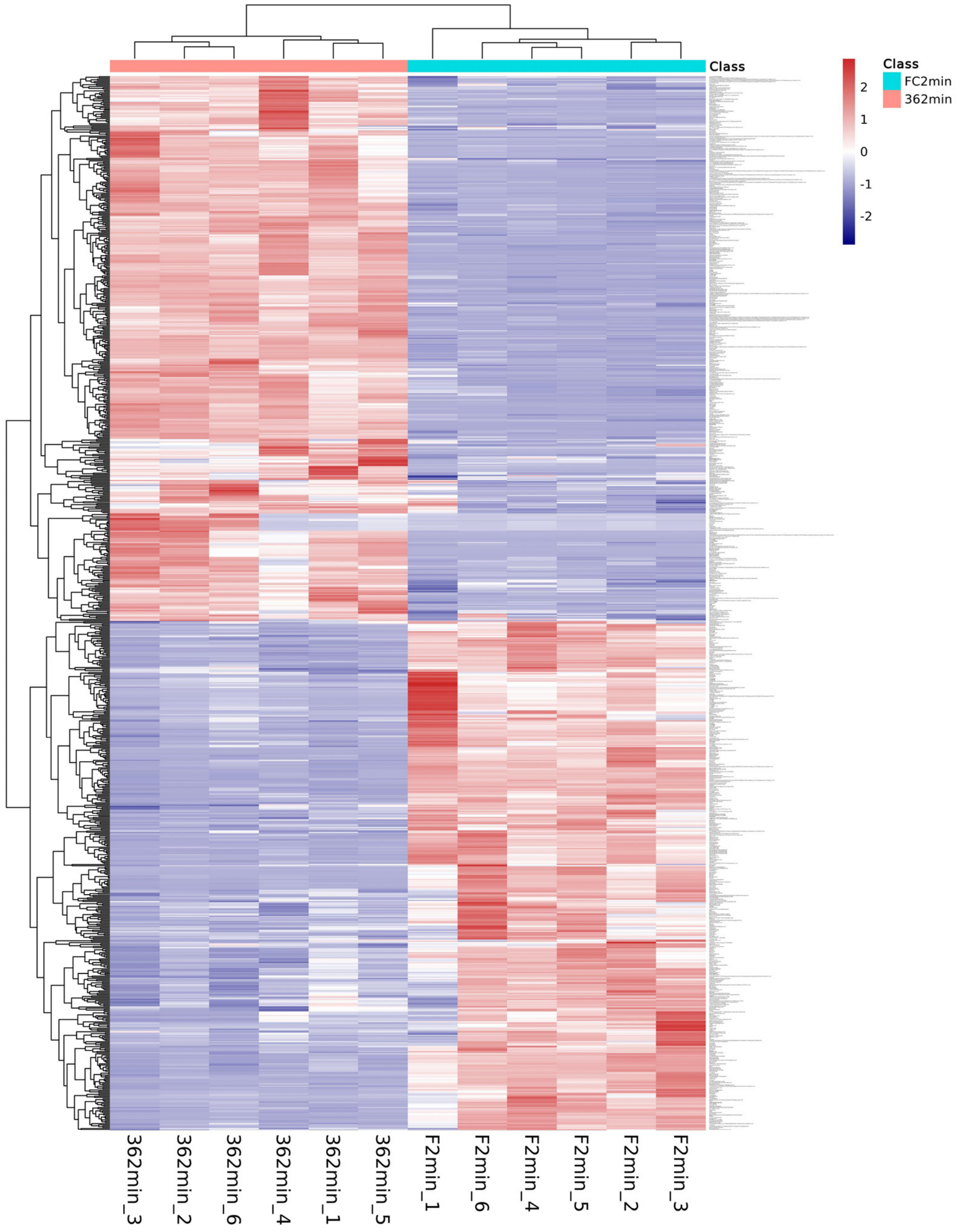

Class
Class
FC2min
362min
2
1
0
-1
-2
362min_3
362min_2
362min_6
362min_4
362min_1
362min_5
F2min_1
F2min_6
F2min_4
F2min_5
F2min_2
F2min_3

(**b**)

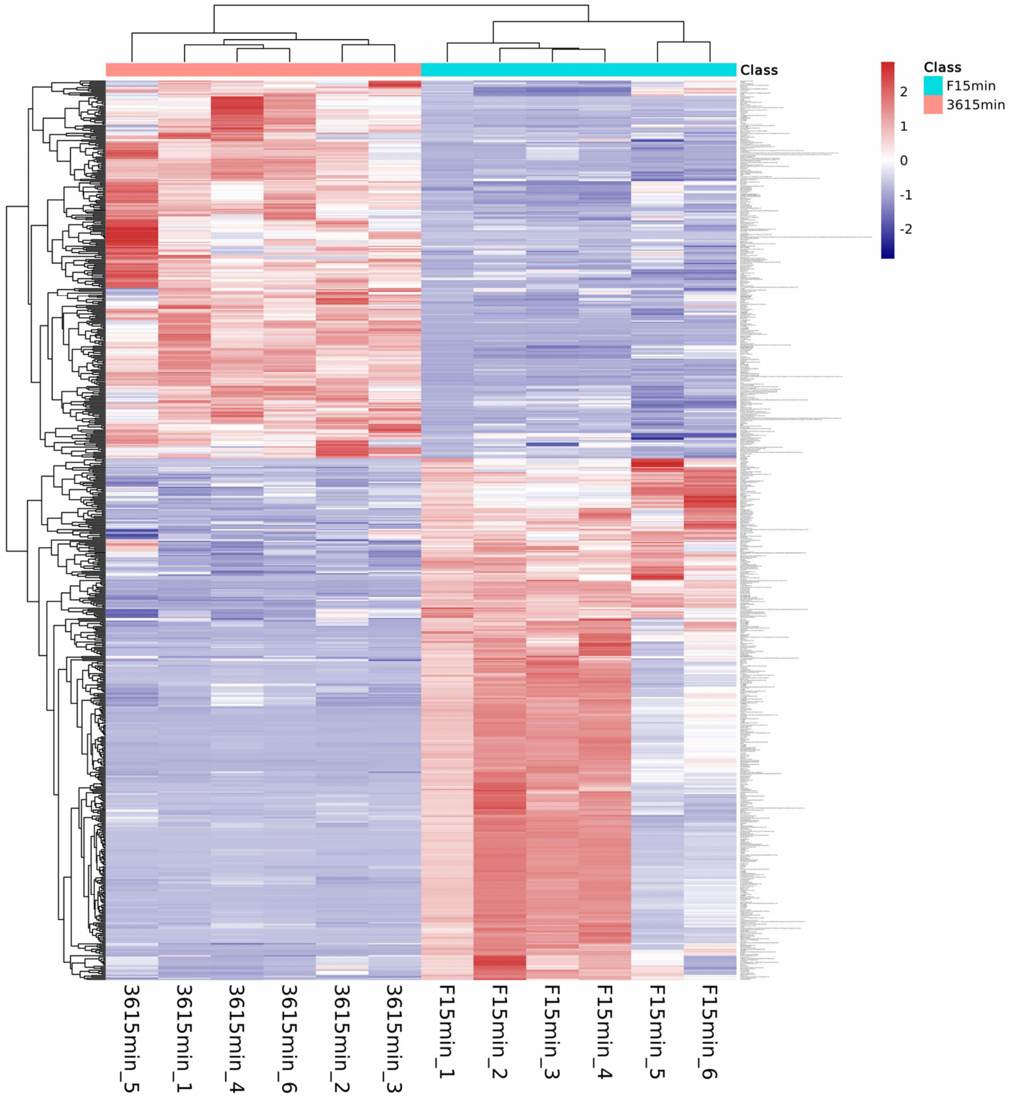
Class
Class
F15min
3615min
2
1
0
-1
-2
3615min_5
3615min_1
3615min_4
3615min_6
3615min_2
3615min_3
F15min_1
F15min_2
F15min_3
F15min_4
F15min_5
F15min_6

(**c**)

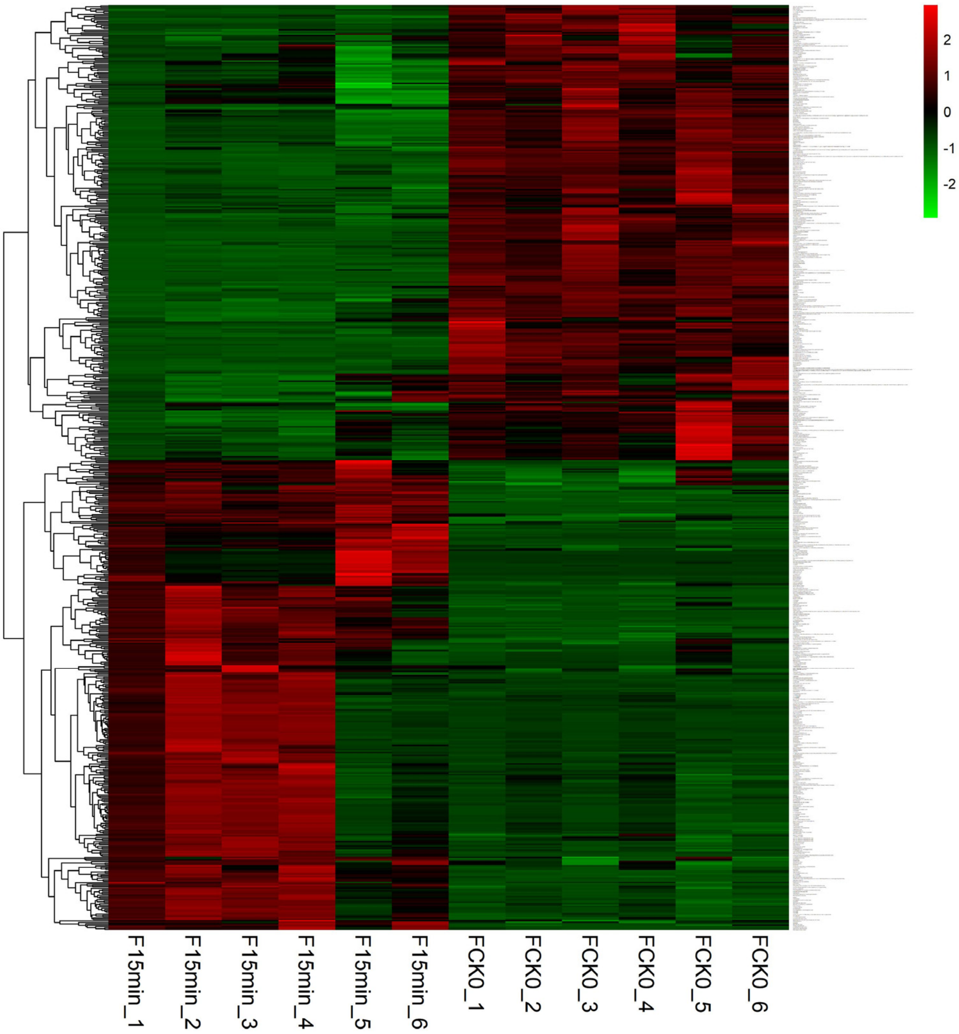
2
1
0
-1
-2
F15min_1
F15min_2
F15min_3
F15min_4
F15min_5
F15min_6
FCK0_1
FCK0_2
FCK0_3
FCK0_4
FCK0_5
FCK0_6

(**d**)

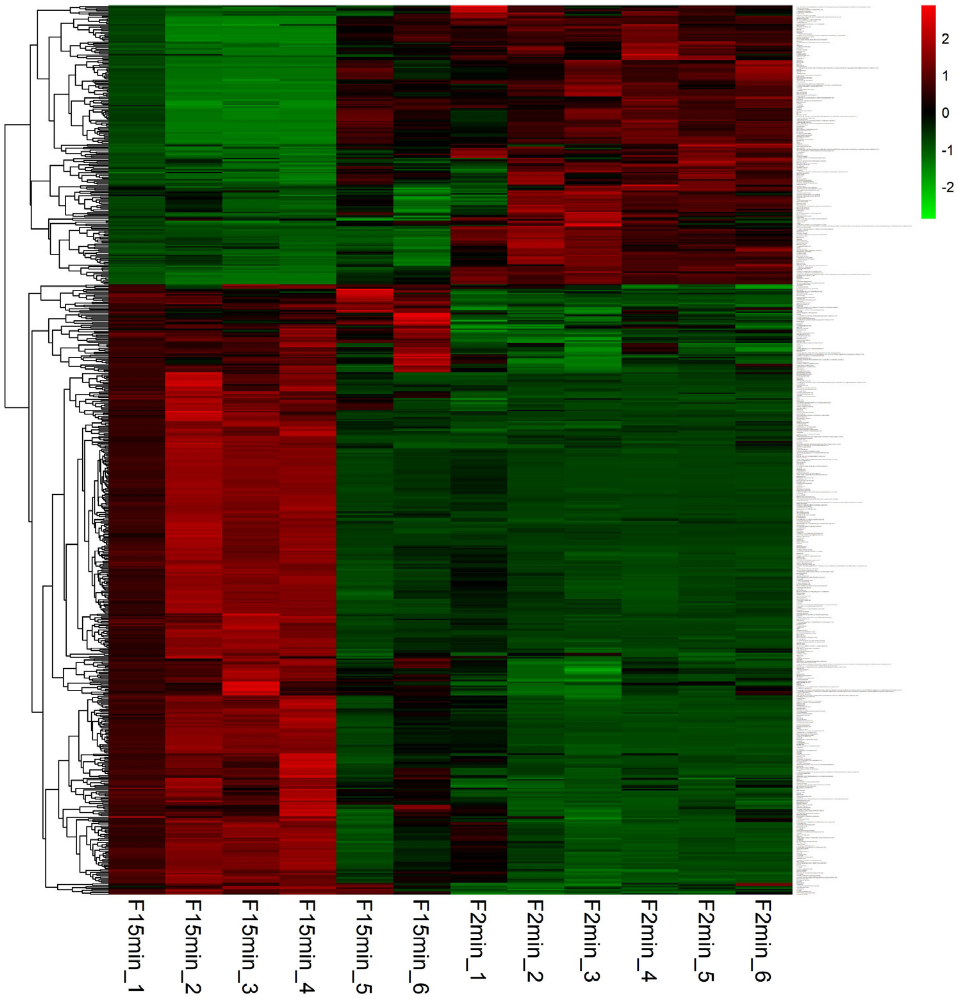
2
1
0
-1
-2
F15min_1
F15min_2
F15min_3
F15min_4
F15min_5
F15min_6
F2min_1
F2min_2
F2min_3
F2min_4
F2min_5
F2min_6

(**e**)

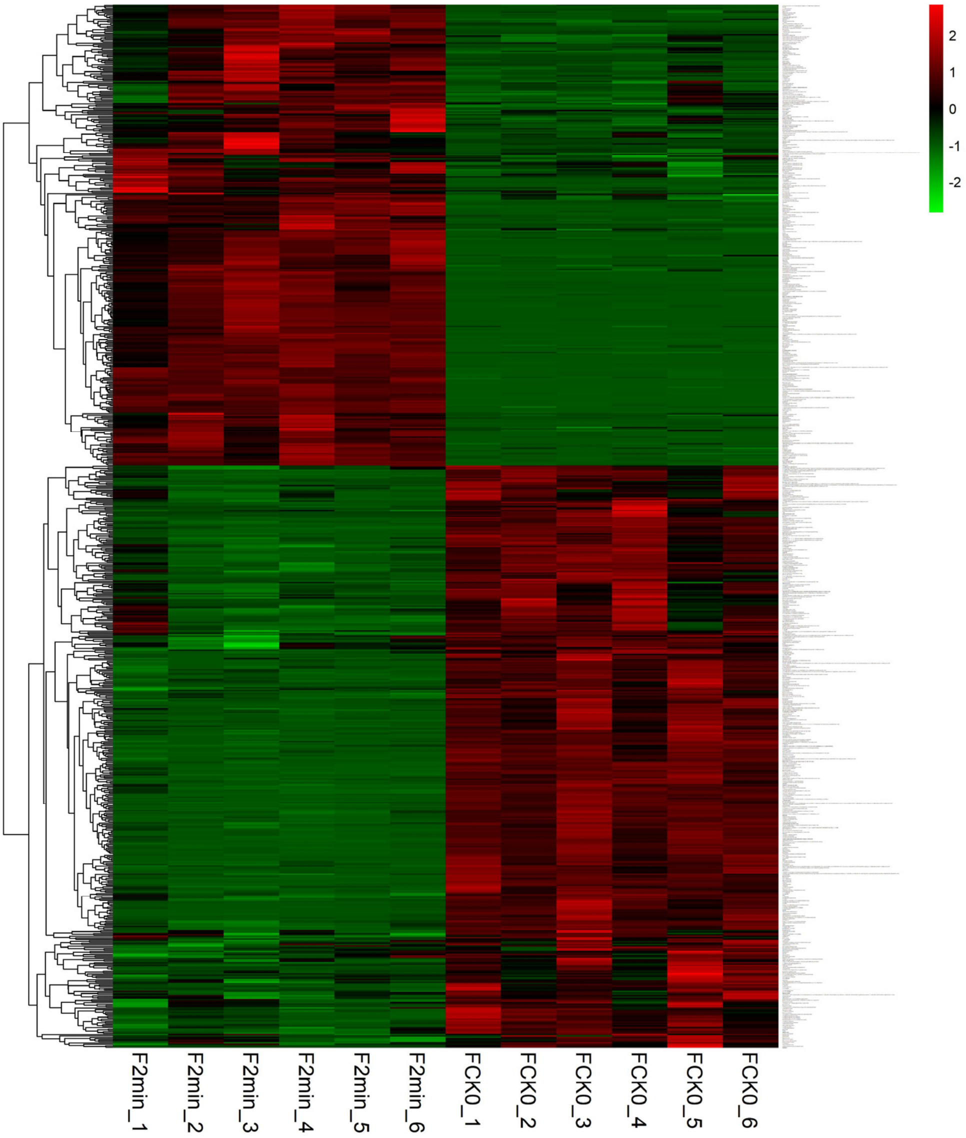
2
1
0
-1
-2
F2min_1
F2min_2
F2min_3
F2min_4
F2min_5
F2min_6
FCK0_1
FCK0_2
FCK0_3
FCK0_4
FCK0_5
FCK0_6

(**f**)

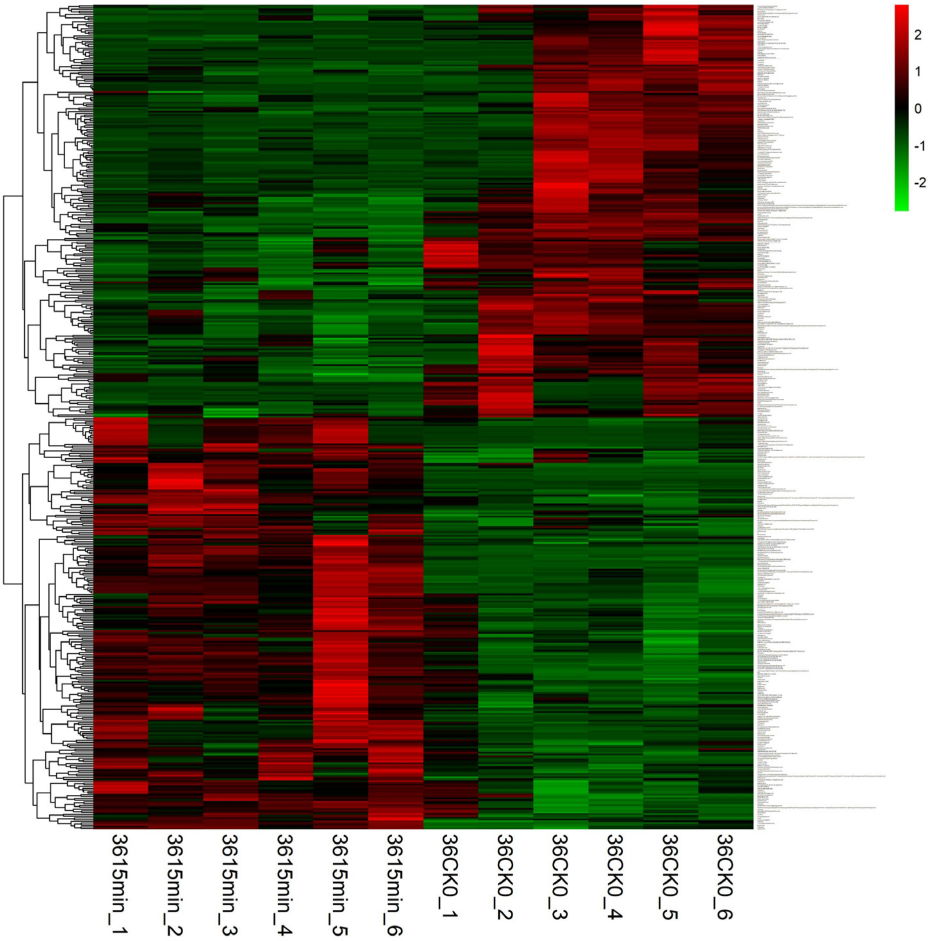
2
1
0
-1
-2
3615min_1
3615min_2
3615min_3
3615min_4
3615min_5
3615min_6
36CK0_1
36CK0_2
36CK0_3
36CK0_4
36CK0_5
36CK0_6

(g)

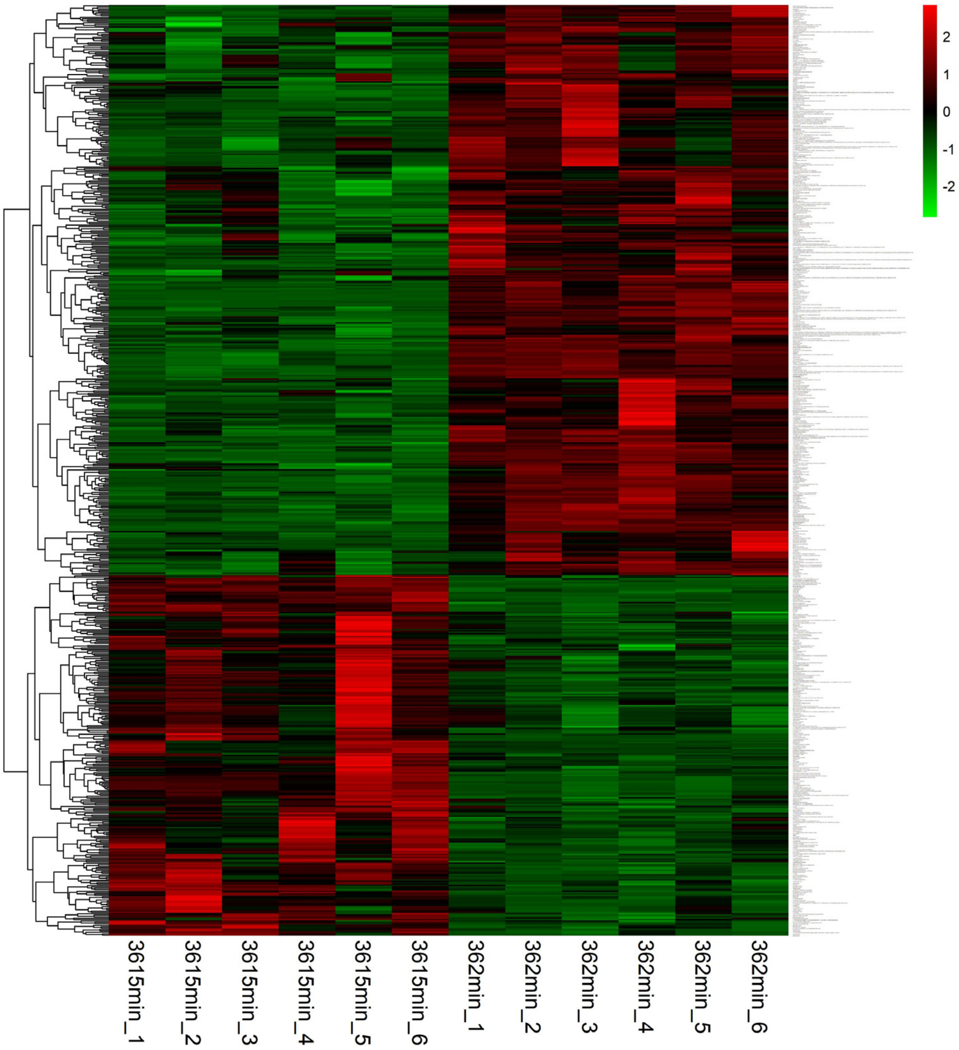
2
1
0
-1
-2
3615min_1
3615min_2
3615min_3
3615min_4
3615min_5
3615min_6
362min_1
362min_2
362min_3
362min_4
362min_5
362min_6

(**h**)

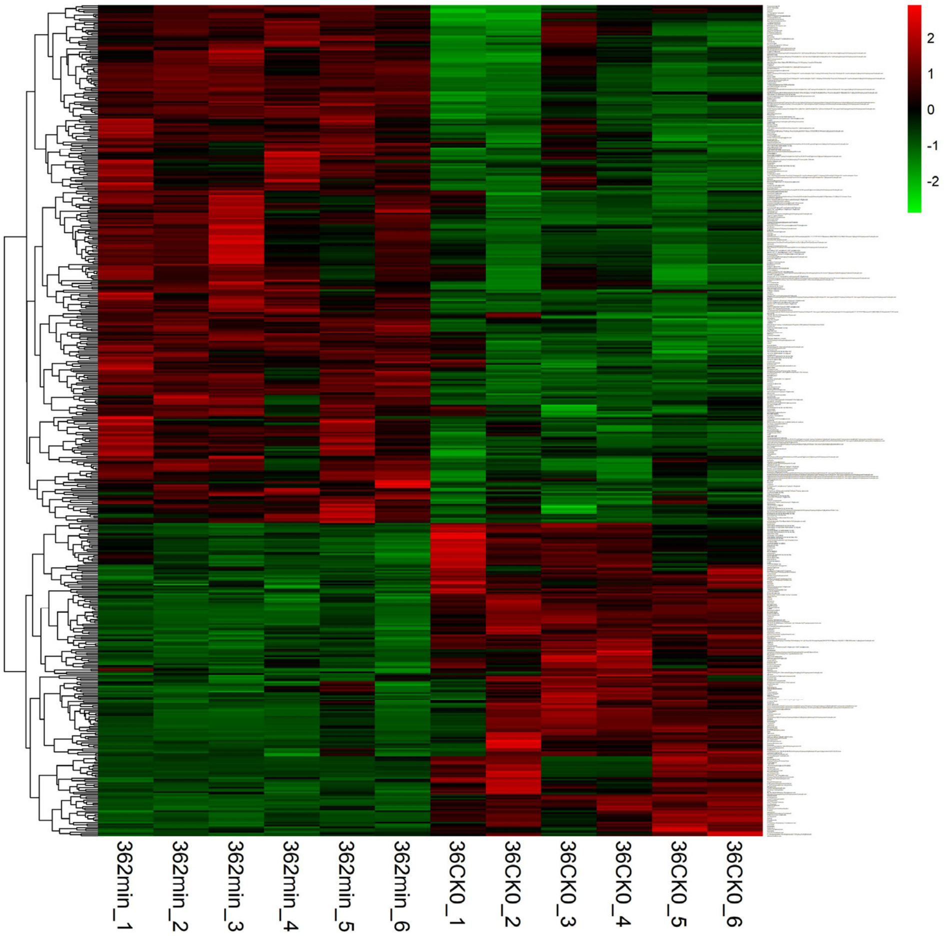

(**i**)

Figure S1: The differential metabolite heat maps of comparison groups. (**a**) F CK0/36 CK0 group; (**b**) F 2 min/36 2 min group; (**c**) F 15 min/36 15 min group; (**d**) F 15 min/F CK0 group; (**e**) F 15 min/F 2 min group; (**f**) F 2 min/F CK0 group; (**g**) 36 15 min/36 CK0; (**h**) 36 15 min/36 2 min; (**i**) 36 2 min/36 CK0.

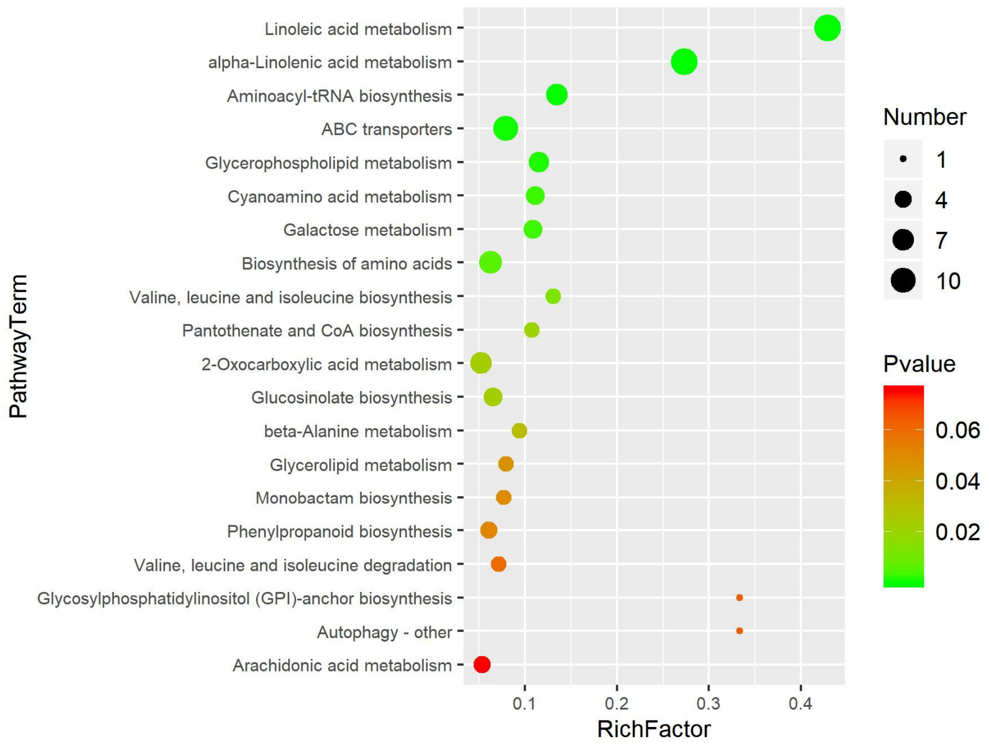
Linoleic acid metabolism
alpha-Linolenic acid metabolism
Aminoacyl-tRNA biosynthesis
ABC transporters
Glycerophospholipid metabolism
Cyanoamino acid metabolism
Galactose metabolism
Biosynthesis of amino acids
Valine, leucine and isoleucine biosynthesis
Pantothenate and CoA biosynthesis
2-Oxocarboxylic acid metabolism
Glucosinolate biosynthesis
beta-Alanine metabolism
Glycerolipid metabolism
Monobactam biosynthesis
Phenylpropanoid biosynthesis
Valine, leucine and isoleucine degradation
Glycosylphosphatidylinositol (GPI)-anchor biosynthesis
Autophagy - other
Arachidonic acid metabolism
PathwayTerm
0.1
0.2
0.3
0.4
RichFactor
Number
1
4
7
10
Pvalue
0.06
0.04
0.02

(**a**)

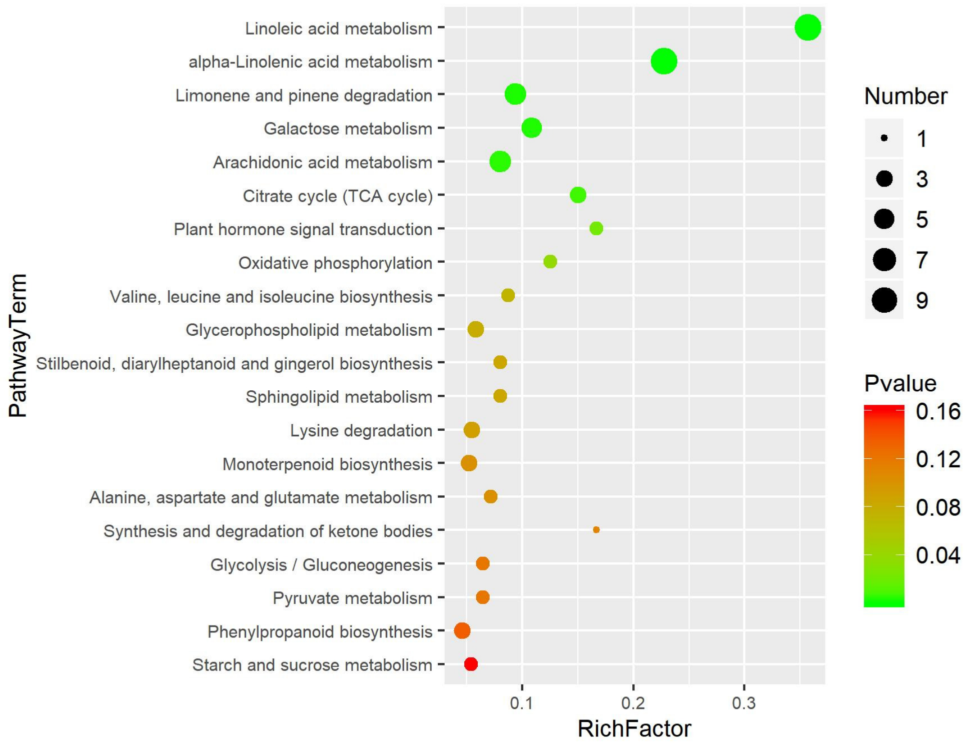
Linoleic acid metabolism
alpha-Linolenic acid metabolism
Limonene and pinene degradation
Galactose metabolism
Arachidonic acid metabolism
Citrate cycle (TCA cycle)
Plant hormone signal transduction
Oxidative phosphorylation
Valine, leucine and isoleucine biosynthesis
Glycerophospholipid metabolism
Stilbenoid, diarylheptanoid and gingerol biosynthesis
Sphingolipid metabolism
Lysine degradation
Monoterpenoid biosynthesis
Alanine, aspartate and glutamate metabolism
Synthesis and degradation of ketone bodies
Glycolysis / Gluconeogenesis
Pyruvate metabolism
Phenylpropanoid biosynthesis
Starch and sucrose metabolism
PathwayTerm
0.1
0.2
0.3
RichFactor
Number
1
3
5
7
9
Pvalue
0.16
0.12
0.08
0.04

(**b**)

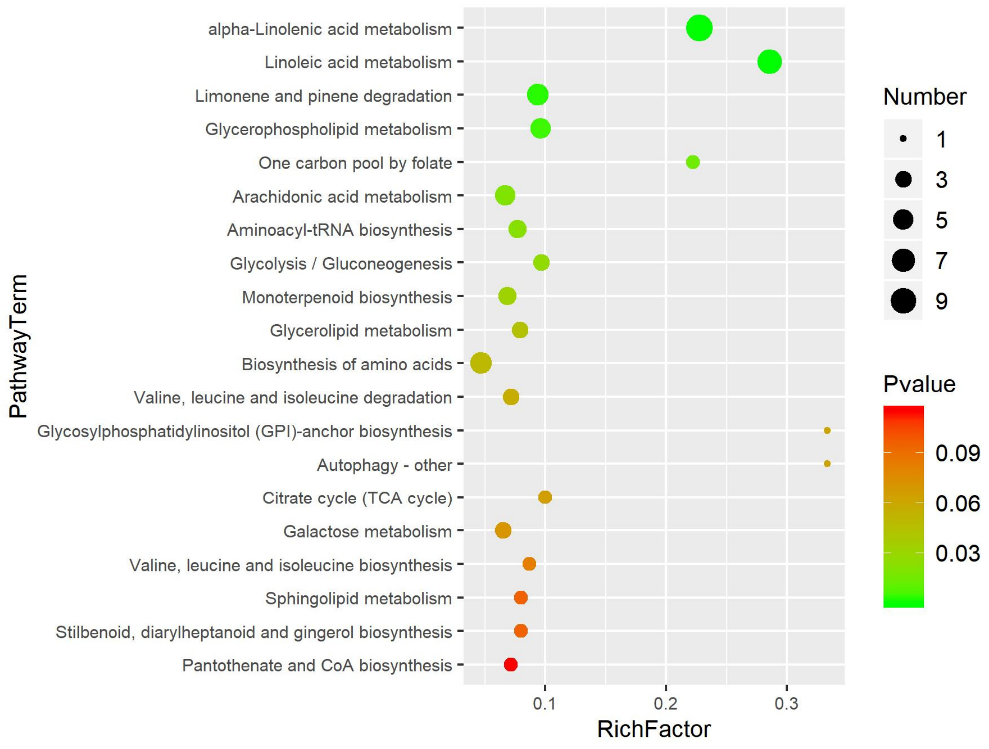
alpha-Linolenic acid metabolism
Linoleic acid metabolism
Limonene and pinene degradation
Glycerophospholipid metabolism
One carbon pool by folate
Arachidonic acid metabolism
Aminoacyl-tRNA biosynthesis
Glycolysis / Gluconeogenesis
Monoterpenoid biosynthesis
Glycerolipid metabolism
Biosynthesis of amino acids
Valine, leucine and isoleucine degradation
Glycosylphosphatidylinositol (GPI)-anchor biosynthesis
Autophagy - other
Citrate cycle (TCA cycle)
Galactose metabolism
Valine, leucine and isoleucine biosynthesis
Sphingolipid metabolism
Stilbenoid, diarylheptanoid and gingerol biosynthesis
Pantothenate and CoA biosynthesis
PathwayTerm
0.1
0.2
0.3
RichFactor
Number
1
3
5
7
9
Pvalue
0.09
0.06
0.03

(c)

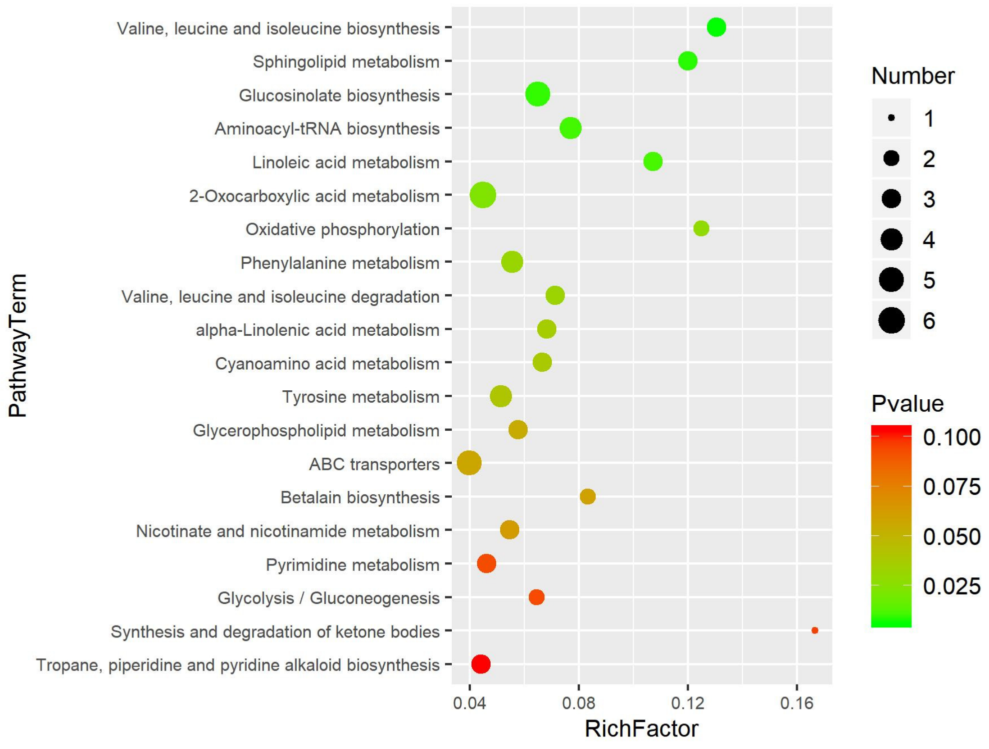
Valine, leucine and isoleucine biosynthesis
Sphingolipid metabolism
Glucosinolate biosynthesis
Aminoacyl-tRNA biosynthesis
Linoleic acid metabolism
2-Oxocarboxylic acid metabolism
Oxidative phosphorylation
Phenylalanine metabolism
Valine, leucine and isoleucine degradation
alpha-Linolenic acid metabolism
Cyanoamino acid metabolism
Tyrosine metabolism
Glycerophospholipid metabolism
ABC transporters
Betalain biosynthesis
Nicotinate and nicotinamide metabolism
Pyrimidine metabolism
Glycolysis / Gluconeogenesis
Synthesis and degradation of ketone bodies
Tropane, piperidine and pyridine alkaloid biosynthesis
PathwayTerm
0.04
0.08
0.12
0.16
RichFactor
Number
1
2
3
4
5
6
Pvalue
0.100
0.075
0.050
0.025

(d)

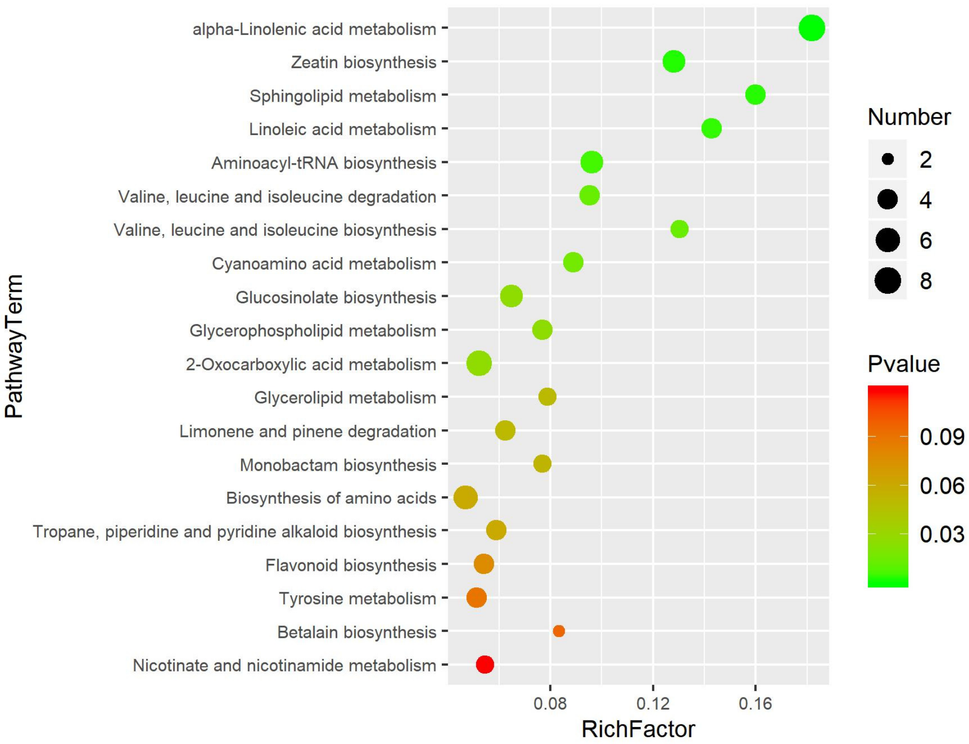

(**e**)

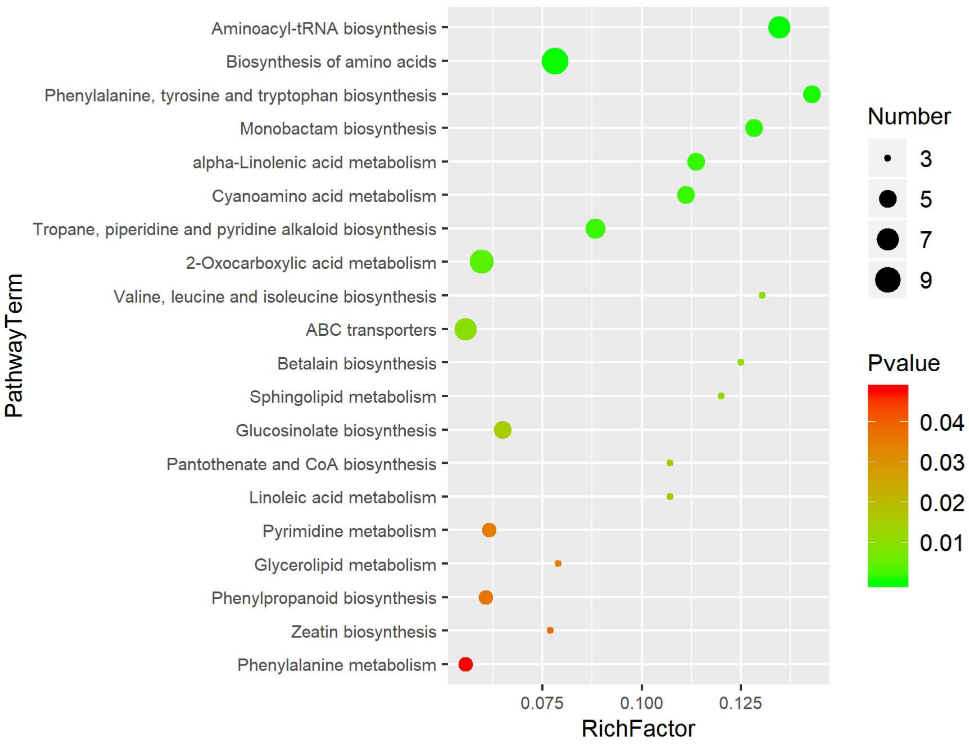

(**f**)

Figure S2: The differential metabolite bubble maps of comparison groups. (**a**) F 15 min/F CK0 group; (**b**) F 15 min/F 2 min, group. (**c**) F 2 min/F CK0 group; (**d**) 36 15 min/36 CK0; (**e**) 36 15 min/36 2 min; (**f**) 36 2 min/36 CK0.
